# The potential of halophilic and halotolerant bacteria for the production of antineoplastic enzymes: L-asparaginase and L-glutaminase

**DOI:** 10.17179/excli2016-146

**Published:** 2016-04-18

**Authors:** Pejman Shirazian, Sedigheh Asad, Mohammad Ali Amoozegar

**Affiliations:** 1 Department of Biotechnology, College of Science, University of Tehran, Tehran, Iran; 2 Extremophiles Laboratory, Department of Microbiology, School of Biology and Center of Excellence in Phylogeny of Living Organisms, College of Sciences, University of Tehran

**Keywords:** L-asparaginase, L-glutaminase, halophilic bacteria, therapeutic enzymes, response surface methodology

## Abstract

L-asparaginase and L-glutaminase can be effectively used for the treatment of patients who suffer from accute lymphoblastic leukemia and tumor cells. Microbial sources are the best source for the bulk production of these enzymes. However, their long-term administration may cause immunological responses, so screening for new enzymes with novel properties is required. Halophilic and halotolerant bacteria with novel enzymatic characteristics can be considered as a potential source for production of enzymes with different immunological properties. In this study, L-asparaginase and L-glutaminase production by halophilic bacteria isolated from Urmia salt lake was studied. Out of the 85 isolated halophilic and halotolerant bacterial strains, 16 (19 %) showed L-asparaginase activity and 3 strains (3.5 %) showed L-glutaminase activity. Strains with the highest activities were selected for further studies. Based on 16S rDNA sequence analysis, it was shown that the selected isolates for L-asparaginase and L-glutaminase production belong to the genus *Bacillus *and *Salicola*, respectively. Both enzymes were produced extracellularly. The strain with the most L-asparaginase production did not show L-glutaminase production which is medically important. The effects of key parameters including temperature, initial pH of the solution, and concentrations of glucose, asparagine or glutamine, and sodium chloride were evaluated by means of response surface methodology (RSM) to optimize enzymes production. Under the obtained optimal conditions, L-asparaginase and L-glutaminase production was increased up to 1.5 (61.7 unit/mL) and 2.6 fold (46.4 unit/mL), respectively.

## Introduction

For many years cancer therapy has relied essentially on antiproliferative agents like low molecular weight chemicals and radiotherapy. The former acts by rapidly killing reproducing cells. However, the application of radiotherapy is restricted to the treatment of solid tumors in the absence of metastasis. Therefore, there is an important need for treatments with enhanced specificity in the cancer therapy (Pasut et al., 2008[22]).

High affinity, specificity and catalytic efficiency are the important features of enzymes that distinguish them from all other types of drugs. Therapeutic enzymes (both digestive and metabolic) can be used medically for the treatment of various diseases such as cancer, cystic fibrosis, inflammation and digestive disorders (Kaur and Sekhon, 2012[13]; Vellard, 2003[32]). Various therapeutic enzymes like L-asparaginase, L-arginase, L-tyrosinase, L-glutaminase, α- and β-glucosidase and β-galactosidase have been used in cancer treatments (Bar, 1970[1]). The sources of these enzymes include animals, plants, bacteria and fungi (Bar, 1970[1]; Sabu et al., 2000[27]).

Certain tumour cells and leukemic lymphoblasts require an external source of L-asparagine for growth and multiplication since they lack or have very low level of asparagine synthetase, an enzyme normally expressed in healthy cells (Gulati et al., 1997[9]; Kidd, 1953[14]; Pasut et al., 2008[22]). Therefore, asparaginase (L-asparagine amidohydrolase, EC3.5.1.1), the enzyme that catalyzes the hydrolysis of L-asparagine into L-aspartic acid and ammonia, can selectively kill tumor cells that rely on asparagine supplied by the serum for survival (Gulati et al., 1997[9]). The potential of asparaginase in cancer treatment was first reported in 1953 (Kidd, 1953[14]). Nowadays L-asparaginase from *E. coli* and *Erwinia *sp. is used as anti-tumor and anti-leukemia agent (Gulati et al., 1997[9]; Peterson and Ciegler, 1969[23]). As found for other commonly non-essential amino acids, some tumors are auxotrophic for glutamine because of their incomplete enzymatic pool. Therefore, glutamine-depleting enzymes can be useful against certain tumors. Moreover to protein synthesis, glutamine is required for DNA synthesis (Roberts et al., 2001[26]). L-glutaminase is used in the treatment of cancer (Roberts et al., 1970[25], 2001[26]) and HIV (Schmid and Roberts, 1974[29]). The medical use of therapeutic enzymes, is limited because of the immunological responses in their long-term administration; so new enzymes with novel immunological properties are required (Howard and Carpenter, 1972[10]; Kidd, 1953[14]; Sarquis et al., 2004[28]). Halophilic and halotolerant bacteria have shown great potential in the production of enzymes with novel characteristics in the detergent (Izotova et al., 1983[12]), baking (Coronado et al., 2000[3]), dairy (Müller-Santos et al., 2009[19]), and leather industries (Vidyasagar et al., 2006[33]); however there are few reports regarding their enzymes as therapeutic agents.

The aim of this work was to study the potential of this group of bacteria for the production of therapeutic enzymes. A quantitative assay was performed with the goal of selecting bacteria with efficient L-asparaginase and L-glutaminase production. The selected strains were characterized by phenotypic description and phylogenetic analysis based on 16S rDNA sequence comparisons. After the preliminary screening tests to show the most effective environmental factors on enzyme production, response surface methodology (RSM) was applied to optimize the conditions for maximum yield. 

## Materials and Methods

### Chemicals

L-asparagine, L-glutamine, phenol red, bromothymol blue and glucose were purchased from Sigma-Aldrich (St. Louis, MO, USA). Taq DNA polymerase and dNTP were obtained from Fermentas (Burlington, Canada) and Stratagene (La Jolla, USA), respectively. Molecular biology kits were from Fermentas (Burlington, Canada). Primers were supplied by Alpha DNA (Montreal, Canada). All other chemicals were obtained from Merck (Darmstadt, Germany). All chemicals were of analytical grade purity.

### Sampling and culture conditions

Water samples were collected in sterile collection tubes from different parts of the Urmia salt lake. Samples were cultured in saline nutrient agar medium containing NaCl (81 g/l), MgSO_4_.7H_2_O (9.7 g/l), MgCl_2_.H_2_O (7.0 g/l), CaCl_2_ (3.6 g/l) and KCl (2.0 g/l). The pH was adjusted to 7.0. Cultures were incubated at 37 °C for 72 hours. Isolated strains were purified by plate streaking technique on nutrient agar supplemented with the mentioned salts.

### L-asparaginase and L-glutaminase production

Screening for L-asparaginase and L-glutaminase production was performed by the method of rapid plate assay (Gulati et al., 1997[9]). Modified M-9 medium containing: Na_2_HPO_4_.2H_2_O, 6.0 g; KH_2_PO_4_, 3.0 g; NaCl, 20.5 g; L-asparagine or L-glutamine, 5.0 g; MgSO_4_.7H_2_O, 0.5 g; CaCl_2_.2H_2_O, 0.15 g; Glucose, 2.0 g and agar, 15.0 g in 1000 mL of distilled water (pH 7.0), was prepared. 2.5 mL of 3 % (w/v) stock solution of phenol red or bromothymol blue in ethanol was added to the media as pH indicator. Samples were incubated at 37 °C for 48 hours. The indicator's color change appeared around bacterial colonies after 24 and 48 hours were used as a sign for L-asparaginase or L-glutaminase production.

### Identification of the isolates

The morphological and physiological characterization of the selected isolates were performed in nutrient broth containing 5 % (w/v) NaCl and were tested as described by Smibert and Krieg (1994[30]). The genomic DNA of the two selected strains was extracted with the Fermentas DNA extraction kit, following the manufacturer's recommended procedure. The 16S rDNA genes were amplified using 27F (5ʹ-AGAGTTTGATYMTGGCTCAG-3ʹ) and 1492R (5ʹ-AAGGAGGTGATCCAGCCGCA-3ʹ) universal primers. The PCR reaction conditions included initial denaturation at 94 °C for 5 min, 35 cycles including 94 °C for 30 s, 57 °C for 1 min, 72 °C for 100 s and final extension at 72 °C for 15 min. The purified PCR products were sequenced in both directions using an automated sequencer by Macrogen (Korea). The sequence similarity searches were done using the BLAST program, available from the National Centre for Biotechnology Information (NCBI).

### Enzyme assay

Enzyme activity was determined by nesslerization for both enzymes (Gulati et al., 1997[9]; Roberts et al., 2001[26]). Isolated strains were inoculated in modified M-9 broth medium at 37 °C for 24 hours. Cell free crude enzyme was prepared by centrifugation at 5000×g for 20 min. 0.5 ml of supernatant, 1.0 ml of 0.1 M sodium acetate buffer (pH 8.5) and 0.5 ml of 0.05 M L-asparagine or L-glutamine solution were mixed and incubated at 37 °C for 10 min. The reaction was stopped by addition of trichloroacetic acid. The precipitated proteins were removed by centrifugation (10000×g for 20 min). The amount of ammonia in supernatant was determined by nesslerization and expressed as U.mL^-1^. Nessler's reagent was prepared by adding 45.5 g HgI_2_ and 35.0 g KI to 1 liter distilled water containing 112g KOH. 0.5 ml of Nessler's reagent was added to the supernatant and the absorbance was determined at 505 nm using a Perkin Elmer lambda 25 UV/VIS spectrophotometer. One unit (U) of enzyme activity is defined as the amount of enzyme which catalyzes the formation of 1 μmole of ammonia in 1 min at the conditions of assay. Different concentrations of ammonium sulfate ((NH_4_)_2_SO_4_) were used as standards.

### Optimization of enzyme production

#### One-Factor-at-a-Time Method

A set of different culture conditions was used to determine factors with significant effect on the enzyme production. In this set of experiments, the level of each factor was changed, while all other experimental factors remained constant. In this case, different production media were prepared by varying the concentration of carbon, nitrogen and salt sources in the culture media. The effect of pH (4.0 to 8.0), aeration and incubation temperature (28 to 42 °C) were also studied.

#### Response surface methodology (RSM)

Based on the results obtained at previously explained experiments, RSM was applied to optimize the enzyme production condition and estimating the influence of the selected variables. All experiments were performed in triplicate and the results were analyzed by the Design Expert software (version 7.0, Stat-Ease, Inc., Minneapolis, MN). The conditions for enzyme production were optimized using a face centered central composite design (CCD). Moreover, the accuracy of the model was verified by comparing the model predictions with the experimental data which were not included in the model estimation. The optimum values of the chosen variables were obtained by calculating the regression equation, besides analyzing the response surface contour plots.

## Results

Being intrinsically stable and active at high salt concentrations, halophilic and halotolerant enzymes offer important opportunities in biotechnological applications, such as food processing, bioremediation and biosynthetic processes. Hence, the finding of novel enzymes showing optimal activities at various ranges of salt concentrations, temperatures and pH values is of great importance. In the present research we have studied the potential of halophilic and halotolerant bacteria for the production of l-asparaginase and l-glutaminase as antineoplastic enzymes. Total of 85 halotolerant bacterial strains were isolated from environmental samples (Urmia lake). Primary physiological tests were performed to differentiate the isolated strains (Table 1(Tab. 1)). It was shown that among the isolated strains, 71 isolates were Gram-stain-positive and 14 were Gram-stain-negative. All strains were screened for L-asparaginase and L-glutaminase production. It was observed that 16 strains (19 %) have L-asparaginase activity and only 3 strains (3.5 %) have L-glutaminase activity. All the L-glutaminase producers and 13 of the L-asparaginase producers were Gram-positive. Quantitative enzyme activity measurements were done in broth media to find the efficient bacteria (Table 1(Tab. 1)). The results indicated that strains with the most L-asparaginase activity, namely gA5, gb2 and MB10 have no L-glutaminase activity. 

**Figure 1:**
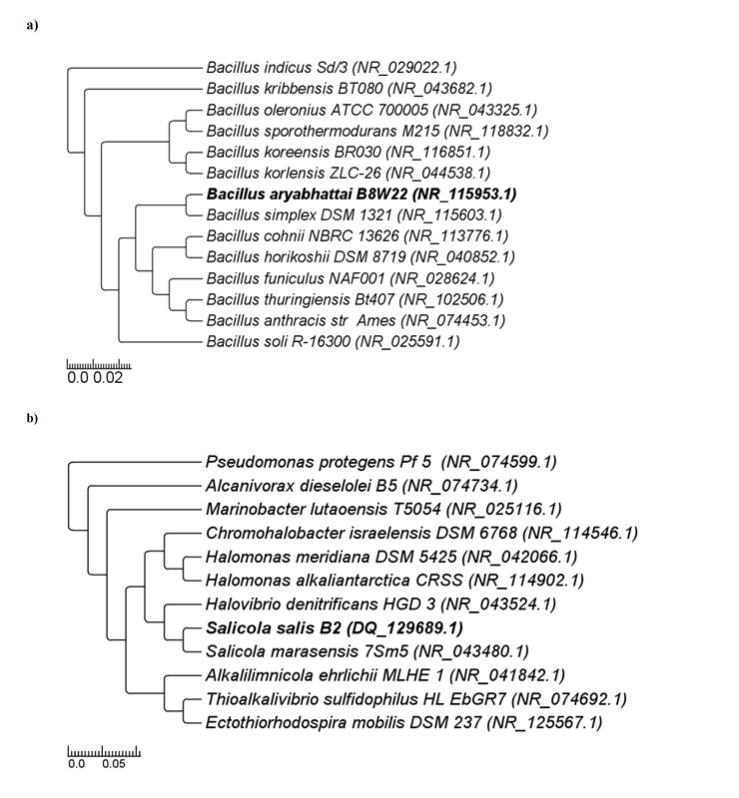
Phylogenetic tree of selected producer strains Neighbor-joining phylogenetic tree based on 16S rRNA gene sequences, showing the position of strain a) gA5, b) F14 and the related genera. GenBank accession numbers are given in parentheses. Bootstrap values (%) are based on 1000 replicates.

Strains gA5 and F14, respectively with the most L-asparaginase and L-glutaminase production, were selected for further studies. The alignment of the amplified 16S rRNA gene fragments showed that gA5 and F14 strains have 100 % and 99.87 % similarity to *Bacillus aryabhattai* and *Salicola salis*, respectively (Figure 1(Fig. 1)). 16S rRNA gene sequences were deposited in the NCBI database under GenBank accession numbers EF114313 and DQ129689.

**Figure 2:**
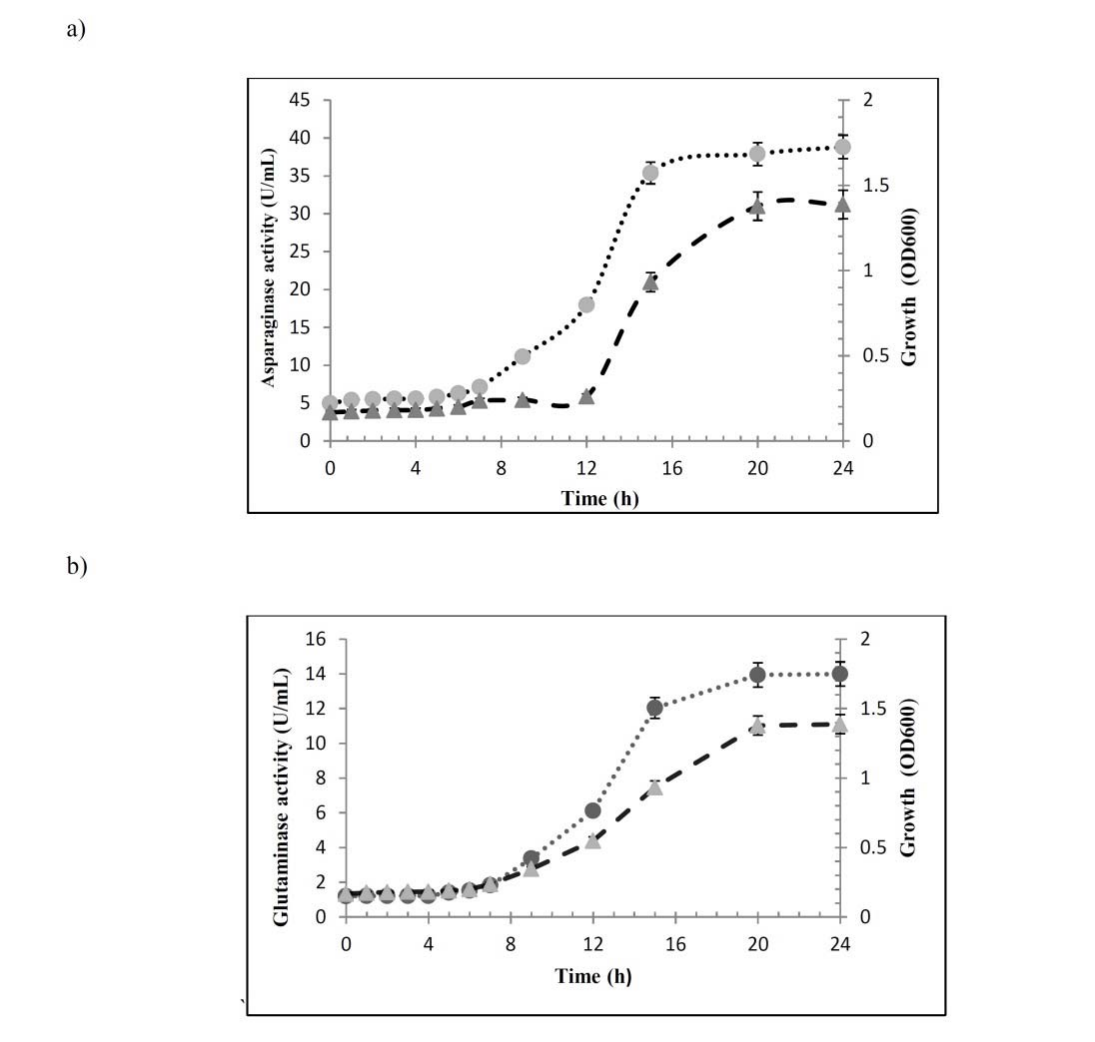
Correlation between bacterial growth and enzyme production. Growth curve and enzyme production of a) gA5 strain, b) F14 strain. ●:enzyme activity,▲:bacterial growth

Comparison of the growth curve and enzyme production in selected isolates showed that both enzymes were produced along with the bacterial growth (Figure 2(Fig. 2)). Examining the cell extracts and culture supernatants for L-asparaginase or L-glutaminase activity, showed that both enzymes were secreted to the culture media. 

In order to optimize the enzyme production, first the influence of different factors was checked by one-factor-at-a-time method. It was shown that temperature, pH, concentrations of sodium chloride, glucose, asparagine or glutamine (respectively for L-asparaginase and L-glutaminase production) had significant effects on production of both enzymes. They were selected for further optimization. A central composite design (CCD) was employed to maximize the enzyme production and examine the combined effect of the variables. A total of 50 experimental runs with different combinations of the five factors were carried out. The regression equation was estimated to test all the polynomial models to fit the CCD data. The quadratic model was shown to be as the most appropriate model. 

The results obtained from the central composite design were fitted to a second order polynomial equation to explain the dependence of enzyme production on the medium components. For L-asparaginase: Activity = 

+36.67 - 8.79A + 0.53B - 6.50C + 2.41D + 4.91E - 0.031AB - 0.97AC - 1.09AD - 1.22AE - 0.094BC - 0.094BD - 0.094BE - 1.16CD - 1.28CE - 1.16DE + 0.66A2 - 8.84B2 + 4.66C2 - 9.84D2 - 2.34E2, 

where activity predicted response of L-asparaginase production, A, B, C, D and E, are the coded values of temperature, pH, salt concentration, asparagine concentration and glucose concentration. For L-glutaminase: Activity = 

+33.91 - 6.21A - 3.44B + 2.12C + 0.85D + 6.38E + 1.47AB - 1.53AC + 0.094AD - 0.97AE - 0.97BC - 0.094BD - 0.41BE - 0.094CD + 0.094CE + 0.094DE - 5.81A2 - 2.81B2 - 8.31C2 - 2.81D2 + 1.19E2. 

A, B, C, D and E are the coded values of temperature, pH, salt concentration, glutamine concentration and glucose concentration.

The analysis of variance (ANOVA) was used to quantitatively study the statistical significance of the model and the contribution of each factor in the enzyme production. The *F* value in an ANOVA shows the variation due to the selected factor relative to the variation caused by the experimental error. Therefore the larger the F value, the more significant the corresponding term (Tables 2(Tab. 2) and 3(Tab. 3)). 

The *F* value of 40.3 or 22.6 suggests a significant model which means there is only 0.001 % chance that a model with a large *F* value could be caused by noise. The plot of the experimental responses (enzyme production) versus predicted values were drawn (data not shown). The model's goodness of fit was also checked by determination coefficient (R^2^). In the case of L-asparginase, the value of R^2^ was 0.96 and in the case of L-glutaminase, it was 0.94. The values of R^2^ closer to 1 denotes better correlation between the observed and predicted responses.

Under the obtained optimal condition for L-asparaginase production (28 °C, pH 6.0, 3 % NaCl, 5.8 g/L L-asparagine and 5 g/L glucose), an activity of 61.7 U/mL was achieved which was very close to the predicted value, 62 U/mL. The optimal condition for L-glutaminase production was predicted to be at 29.6 °C, pH 4.1, 8.6 % NaCl, 5.8 g/L L-glutamine and 5 g/L glucose with an enzyme activity of 46.4 U/mL. The predicted enzyme production was experimentally checked and the value of 46 U/mL was attained. 

**Figure 3:**
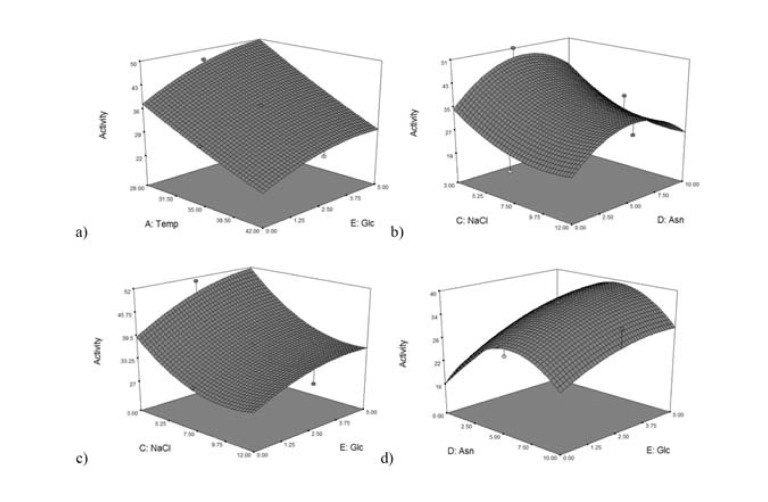
Pair wise combination of significant factors Response surface plot showing the effect of the four variables/experimental factors on L-asparaginase production; a) effect of temperature and glucose, b) effect of NaCl and asparagine, c) effect of NaCl and glucose, d) effect of asparagine and glucose

**Figure 4:**
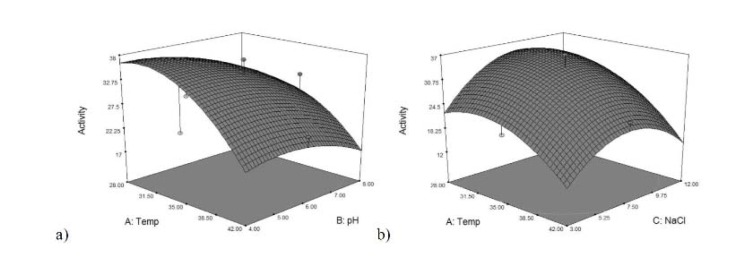
Pair wise combination of significant factors Response surface plot showing the effect of the three variables/experimental factors on L-glutaminase production; a) effect of temperature and pH, b) effect of temperature and NaCl.

Among the interacting effects, temperature-glucose, NaCl-Asparagine, NaCl-glucose and asparagine-glucose were the significant ones in the variable ranges investigated by the model of L-asparaginase production (*p*-values less than 0.0500 indicate model terms are significant). In L-glutaminase production model, temperature-pH and temperature-NaCl were significant. In order to understand these effects, the three dimensional (3D) contour plots of the pair wise combination of significant factors for enzymes production were generated which described predicted response over a range in the design surface (Figures 3(Fig. 3) and 4(Fig. 4)). It was shown that increasing glucose concentration and simultaneously decreasing temperature or NaCl concentration leads to an increase in asparaginase production (Figures 3a and c(Fig. 3)). It could also be concluded that elevated asparagine concentrations (up to 5.8 %) when coupled with an increase in glucose concentration (up to 5 %), improved asparaginase production (Figure 3d(Fig. 3)) but its higher amounts had negative effect. A similar interaction between the NaCl and asparagine concentrations was also observed (Figure 3b(Fig. 3)). In the case of glutaminase production, it could be inferred that decreasing both pH and temperature leads to an increase in enzyme production (Figure 4a(Fig. 4)). It was also shown that rising both NaCl (up to about 6 %) and temperature (up to 35 °C) results in an increase in the glutaminase production yield (Figure 4b(Fig. 4)). At higher values, improvement in enzyme production was negligible. 

## Discussion

One of the major limiting factors for the long-term administration of pharmaceutical enzymes is inducing immunological responses. Therefore, screening for enzymes with novel properties is necessary. Halophilic microorganisms are adapted to survive in ecological niches with high salt concentrations. These microorganisms produce unique biocatalysts but there are few reports assessing their potential for the production of pharmaceutical enzymes. L-asparaginase and L-glutaminase are two of the key enzymes for treatment of leukemic lymphoblasts. In this study, rapid plate assay was used for the screening of halophilc bacterial strains with the potential of L-asparaginase or L-glutaminase production. Asparagine or glutamine consumption releases ammonia which increases pH and will cause indicator's color change from yellow to red (in phenol red) (Gulati et al., 1997[9]) or blue (in bromothymol blue) (Mahajan et al., 2013[17]). Among the 85 screened halophilic strains, two of the best enzyme producers were chosen based on quantitative enzyme activity measurements for further studies. In contrast to L-asparaginase, L-glutaminase production was a rare phenomenon among the studied halophiles. Interestingly the main L-asparaginase producer has shown no glutaminase activity which is a highly desirable characteristic for the cancer therapy (Pasut et al., 2008[22]). The presence of glutaminase impurity may result in cellular stress and neurotoxicity (Nagarajan et al., 2014[20]). There are few reports on glutaminase-free L-asparaginase production by microorganisms such as *Pectobacterium carotovorurm *MTCC 1428 (Kumar et al., 2011[15]), *Vibrio succinogenes* (Distasio et al., 1982[6]), *Pseudomonous stutzeri* (Manna et al., 1995[18]), *Pyrococcus furiosus* and its mutants MTCC 5580-5582 (Kundu et al., 2013[16]).

Another advantage of the isolated strains is their extracellular production of the enzymes. The secretion of the enzymes to the culture media makes the downstream processing easier and cheaper by omitting the cell disruption step in purification process. Moreover moderate halophilic and halotolerant bacteria, unlike halophilic archaea and some extremely halophilic bacteria, do not accumulate inorganic ions (K^+^, Na^+^, Cl^−^) in their cytoplasm to balance the osmotic pressure of the medium (Brown, 1976[2]; Nieto and Vargas, 2002[21]); so their intracellular proteins are not specified for being stable and active in the presence of salts when compared with the extracellular proteins (Essghaier et al., 2014[8]).

The enzyme production was optimized for both producer strains by response surface methodology. The large F values in both cases indicated the significance of the models. 1.5 and 2.6 fold increase in asparaginase and glutaminase production were achieved by varying different environmental factors. The predicted enzyme production was experimentally verified for both enzymes, indicating the model consistency. There are some reports on the L-asparaginase and L-glutaminase production by different bacterial strains, in which 32.3, 51.54 and 135 U/mL of L-asparaginase production were described for *Streptomyces ginsengisoli *(Deshpande et al., 2014[4]), *Bacillus cereus* (Thenmozhi et al., 2011[31]) and *Streptomycetes parvulus* (Rajamanicjam et al., 2011[24]), respectively. However none of the reported bacteria are halophilic or halotolerant. In the study of Ebrahiminezhad et al. (2011[7]), considering L-asparaginase production by halophilic and halotolerant bacteria isolated from Maharloo salt lake, *Bacillus* sp. BCCS 034 was found to produce L-asparaginase extracellularly (1.64 IU/ml supernatant).

Iyer and Singhal (2009[11]) reported 119 U/ mL of L-glutaminase production by a *Providencia* sp. strain. In another report by Dilara and Emine (2014[5]), 13.75 U/mL of the extracellular enzyme production was achieved by the isolated *Hypocrea Jecorina* strain. To the best of our knowledge, so far there are no reports on the the L-glutaminase production by halophilic or halotolerant bacteria.

To conclude, we showed that hypersaline lakes which are inhabited by a vast variety of microbial communities have novel microbial strains with potential applications in different biotechnological fields. Based on screening results, it was shown that halophilic and halotolerant bacteria isolated from Urmia salt lake, have the potential of L-asparaginase and L-glutaminase production. Nevertheless, more studies are needed to explore their potential as antineoplastic enzymes.

## Acknowledgement

We would like to thank the research council of the University of Tehran for the financial support of this research.

## Conflict of interest

There is no conflict of interest and no benefit of any kind will be received either directly or indirectly by the author(s).

## Figures and Tables

**Table 1:**
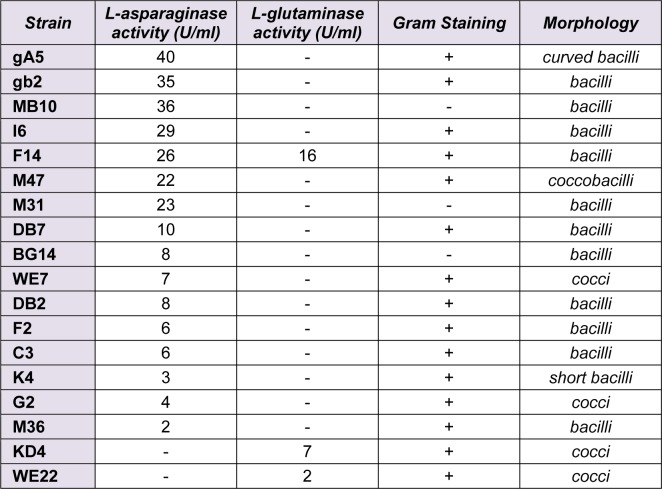
Characteristics of isolated strains with L-asparaginase or L-glutaminase activity

**Table 2:**
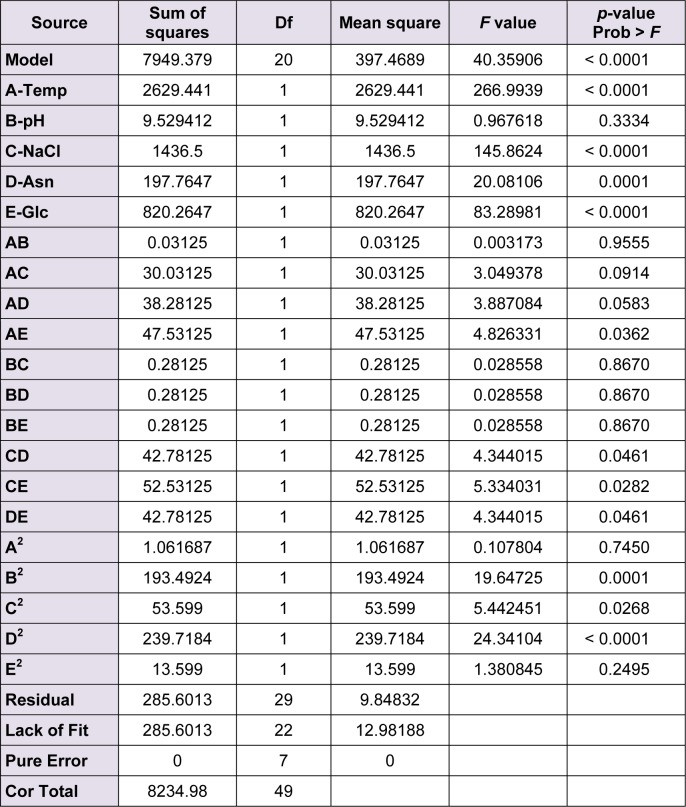
Analysis of variance (ANOVA) for response surface quadratic model for L-asparginase production

**Table 3:**
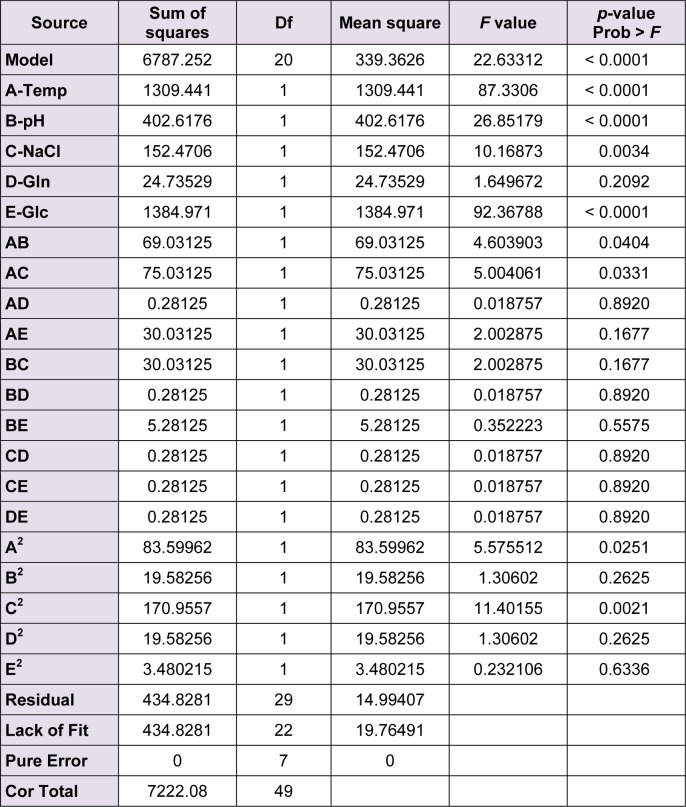
Analysis of variance (ANOVA) for response surface quadratic model for L-glutaminase production
